# Functional Antagonism of WRI1 and TCP20 Modulates *GH3.3* Expression to Maintain Auxin Homeostasis in Roots

**DOI:** 10.3390/plants11030454

**Published:** 2022-02-07

**Authors:** Que Kong, Pui Man Low, Audrey R. Q. Lim, Yuzhou Yang, Ling Yuan, Wei Ma

**Affiliations:** 1 School of Biological Sciences, Nanyang Technological University, Singapore 637551, Singapore; quekong@ntu.edu.sg (Q.K.); puiman001@e.ntu.edu.sg (P.M.L.); limr0029@e.ntu.edu.sg (A.R.Q.L.); yuzhou001@e.ntu.edu.sg (Y.Y.); 2 Kentucky Tobacco Research and Development Center, Department of Plant and Soil Sciences, University of Kentucky, Lexington, KY 40546, USA; lyuan3@uky.edu

**Keywords:** Arabidopsis, gene regulation, protein-protein interaction, transcription factor, WRI1, TCP20

## Abstract

Auxin is a well-studied phytohormone, vital for diverse plant developmental processes. The *GH3* genes are one of the major auxin responsive genes, whose expression changes lead to modulation of plant development and auxin homeostasis. However, the transcriptional regulation of these *GH3* genes remains largely unknown. WRI1 is an essential transcriptional regulator governing plant fatty acid biosynthesis. Recently, we identified that the expression of *GH3.3* is increased in the roots of *wri1-1* mutant. Nevertheless, in this study we found that AtWRI1 did not activate or repress the promoter of *GH3.3* (*proGH3.3*) despite of its binding to *proGH3.3*. Cross-family transcription factor interactions play pivotal roles in plant gene regulatory networks. To explore the molecular mechanism by which WRI1 controls *GH3.3* expression, we screened an Arabidopsis transcription factor library and identified TCP20 as a novel AtWRI1-interacting regulator. The interaction between AtWRI1 and TCP20 was further verified by several approaches. Importantly, we found that TCP20 directly regulates *GH3.3* expression via binding to TCP binding element. Furthermore, AtWRI1 repressed the TCP20-mediated transactivation of *proGH3.3*. EMSAs demonstrated that AtWRI1 antagonized TCP20 from binding to *proGH3.3*. Collectively, we provide new insights that WRI1 attenuates *GH3.3* expression through interaction with TCP20, highlighting a new mechanism that contributes to fine-tuning auxin homeostasis.

## 1. Introduction

The phytohormone auxin (indole-3-acetic acid; IAA) plays a pivotal role in plant developmental processes, such as embryogenesis, organogenesis, shoot and root growth, and organ patterning [1,2,3]. The *Aux/IAA* (AUXIN/INDOLE-3-ACETIC ACID), *SAUR* (small, auxin-induced RNA), and *GH3* (Gretchen Hagen 3) are the major auxin responsive genes [2,4,5]. Changes in *GH3* gene expression affect the plant developmental processes, such as the growth of hypocotyl, root, and shoot [6,7,8,9]. The *GH3* gene family in Arabidopsis comprises 20 members [9]. Several *GH3* genes control the formation of IAA-amino acid conjugates which play roles in storing, transporting, compartmentalizing, and metabolizing auxins [7,10]. These findings suggest that GH3s are essential for mediating auxin homeostasis and auxin-associated growth responses [11,12]. A majority of the *GH3* gene promoters have been found to contain *cis*-acting auxin responsive elements (AuxREs) that are recognized by auxin response factors (ARFs) [2,4,13]. In addition, bZIP transcription factors (e.g., Arabidopsis bZIP11) have been found to bind the G-box-related element (GRE) in the *GH3* promoter to activate *GH3* expression [11,14].

## 2. Results and Discussion

**Figure 1:**
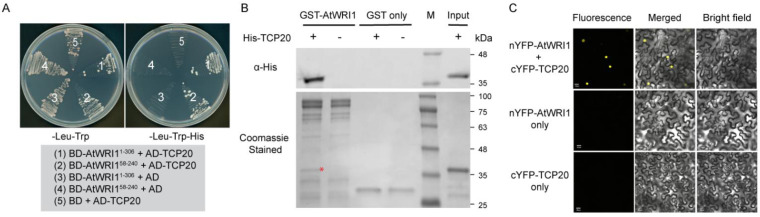
Physical interaction of AtWRI1 with TCP20. (**A**) TCP20 interacts with AtWRI1 variants in yeast cells. Yeast growth on either permissive (-Leu/-Trp) or stringent selective (-Leu/-Trp/-His) medium was shown. The numbers in the plates indicate the co-expression of DNA binding domain (BD) fusions of AtWRI1 with varied amino acid length and activation domain (AD)-TCP20 fusion (indicated in shaded box). (**B**) GST pull-down assay showing the interaction of TCP20 with AtWRI1. The bottom panel shows Coomassie blue stained SDS-PAGE gel of GST pull-down using *E. coli* expressed protein. Red asterisk indicates the pull-down product of His-TCP20 that has identical molecular mass as the purified His-TCP20 in the far-right lane. The upper panel shows the immunoblotting of the pull-down products using anti-His antibody, verifying the presence of the His-TCP20. (**C**) A BiFC assay showing the physical interaction of AtWRI1 with TCP20 in plant cells. Confocal images show *N. benthamiana* epidermal cells transiently co-producing nYFP-AtWRI1 and cYFP-TCP20. Scale bar is 20 μM.

We recently found that the *GH3.3* gene is upregulated in the roots of Arabidopsis *WRINKLED1* (*AtWRI1*) loss-of-function mutant (*wri1-1*) [15]. WRI1 is a member of APETALA2 (AP2) transcription factor family, well known for transcriptional regulation of plant oil accumulation [16,17]. WRI1 functions as a transcriptional activator of genes involved in oil biosynthetic pathways [18,19,20,21]. However, how AtWRI1 regulates the expression of *GH3.3* is unclear. *AtWRI1* is differentially expressed in Arabidopsis embryo over other vegetative tissues. *AtWRI1* also displays significant expression in roots [15,16]. Interestingly, although AtWRI1 is able to bind to *proGH3.3* [15], our dual-luciferase (LUC) transactivation assay showed that AtWRI1 did not activate or repress the promoter of *GH3.3* (*proGH3.3*) (Supplementary Figure S1). We hence hypothesized that AtWRI1 regulates the expression of *GH3.3* through the coordination with an alternative transcriptional regulator. To investigate the molecular mechanism by which WRI1 controls *GH3.3* expression, we performed yeast-two-hybrid (Y2H) assay to screen an Arabidopsis transcription factor library [22], using truncated AtWRI1 variants (AtWRI1^1-306^ and AtWRI1^58-240^) as baits. The truncated AtWRI1 variants as baits are necessary to avoid high transactivation activity mediated by the C-terminus of AtWRI1 [23]. We identified a class I TEOSINTE BRANCHED1/CYCLOIDEA/ PROLIFERATING CELL FACTOR (TCP) family transcription factor TCP20 as a previously unknown interacting partner of AtWRI1. We confirmed that AtWRI1 physically interacts with TCP20 in yeast cells (Figure 1A). In the pull-down assays, His-tagged TCP20 was pulled down by the GST agarose-coupled AtWRI1 (Figure 1B), providing further evidence for the interaction between AtWRI1 and TCP20. To further validate the AtWRI1-TCP20 interaction in vivo, we conducted bimolecular fluorescence complementation (BiFC) assays by co-producing the split YFP fusions of the two proteins, nYFP-AtWRI1 and cYFP-TCP20, in *Nicotiana benthamiana* leaves. We detected YFP fluorescence in leaf samples that co-produced nYFP-AtWRI1 and cYFP-TCP20 (Figure 1C). Taken together, our results verified the physical interaction between AtWRI1 and TCP20. 

**Figure 2:**
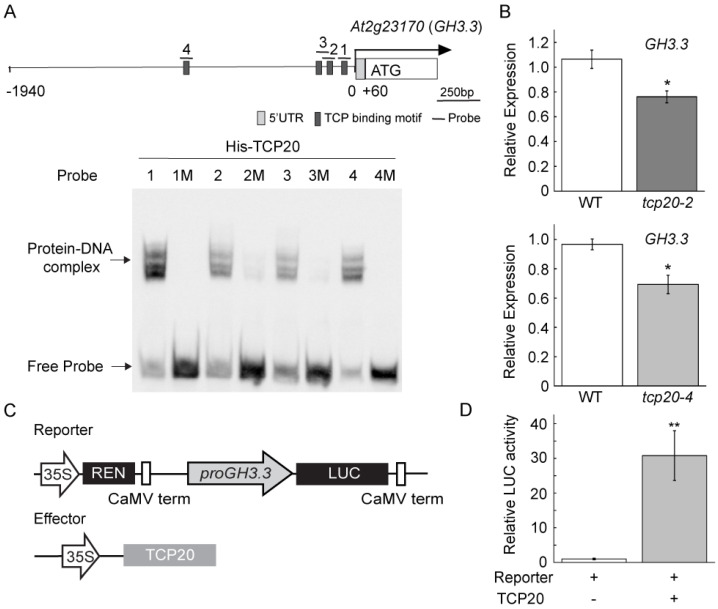
Direct promoter binding and regulation of *GH3.3* by TCP20. (**A**) EMSA showing TCP20 binding to the TCP binding motifs in the *GH3.3* promoter (*proGH3.3*). As indicated in the schematic representation of *proGH3.3,* four biotin-labelled DNA probes (dark boxes indicated by underlined numbers 1–4), containing consensus TCP binding motifs, and their corresponding mutant (M) probes (Supplementary Figure S2) were used for EMSA experiments to test the interaction with purified His-TCP20 protein. The shifted DNA-protein complexes are indicated by an arrow. (**B**) Quantitative real-time PCR (qRT-PCR) analysis of *GH3.3* transcript in the roots of wild-type (WT), *tcp20-2* (SALK_088460), and *tcp20-4* (SALK_041906) mutants. Results are shown as means ± SE (n = 3). “*” indicates a significant difference (*p* < 0.05, Student’s t-test) compared with WT. (**C**) Schematic representation of the constructs used in a transient expression assay in *N. benthamiana* leaves. The *LUC* reporter gene was driven by a 2kb *proGH3.3*. The *Renilla* luciferase (*REN*) reporter gene was controlled by the *CaMV* 35S promoter. (**D**) Transactivation of the *LUC* reporter by TCP20 in *N. benthamiana* leaves. Relative reporter activity in *N. benthamiana,* infiltrated either using the reporter alone or in combination with the effector, was shown. The LUC activity was normalized to the REN activity. Results are shown as means ± SE (n = 5–6). “**” indicates a significant difference (*p* < 0.01, one-way ANOVA) between reporter alone and co-transformation of TCP20 and reporter.

Previous studies show that TCP20 is highly expressed in Arabidopsis roots to regulate numerous genes that are important for plant development and signaling pathways [24,25,26]. However, the molecular mechanism underlying TCP20 working with AtWRI1 to regulate *GH3.3* expression remains unknown. We thus analyzed *proGH3.3* and found four putative TCP binding motifs in the promoter (Supplementary Figure S2). To test whether TCP20 binds to *proGH3.3*, we synthesized four DNA probes (Probe 1–4), each containing an individual putative TCP binding motif with flanking sequences on both sides (Figure 2A and Supplementary Figure S2) and performed electrophoretic mobility shift assay (EMSA) using TCP20 protein. Our result showed that TCP20 bound to all four DNA probes (probe 1–4; Figure 2A); however, TCP20 failed to bind to the DNA probes with nucleotide mutations at the TCP20 binding element (probe 1M–4M; Figure 2A), implying the binding specificity to the TCP motifs in the *proGH3.3*. To explore the transcriptional regulation of *GH3.3* by TCP20, we subsequently characterized two homozygous Arabidopsis *tcp20* T-DNA insertional lines (*tcp20-2* (SALK_088460) and *tcp20-4* (SALK_041906)). We examined the expression of *GH3.3* in roots of *tcp20* loss-of-function mutants and found significant downregulation of *GH3.3* in both lines compared to wild-type (WT) (Figure 2B). We also examined the transactivation of TCP20 on *proGH3.3* using dual-LUC transient expression assay in *N. benthamiana* leaves (Figure 2C). TCP20 significantly activated the *LUC* expression driven by *proGH3.3* in the transient assay (Figure 2D). Taken together, these results indicate that TCP20 directly regulates *GH3.3* expression through binding to the TCP binding motifs in its promoter.

**Figure 3:**
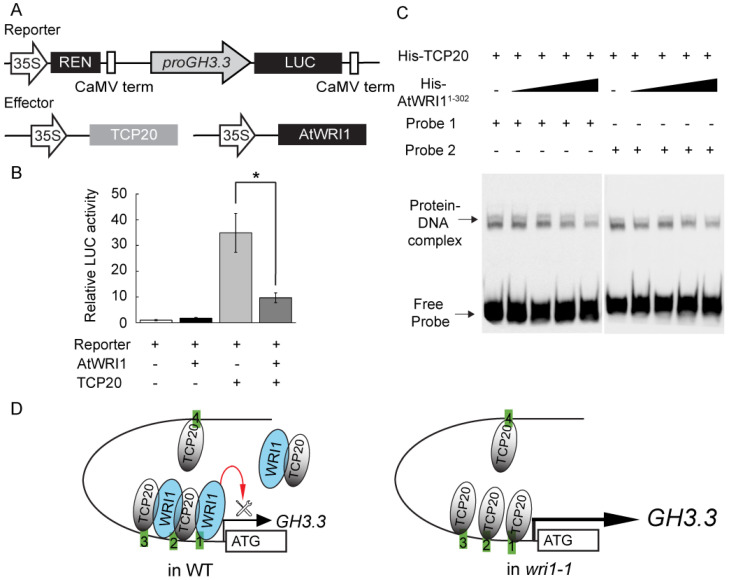
Interference of TCP20 binding to *proGH3.3* by AtWRI1. (**A**) Schematic representation of the constructs used in a transient expression assay in *N. benthamiana*. (**B**) Co-expression of *AtWRI1* with *TCP20* repressed the transactivation activity of TCP20 on *proGH3.3.* Results are shown as means ± SE (n = 5–6). “*” indicates a significant difference (*p* < 0.05, Student’s t-test) between sole expression of *TCP20* and co-expression of *AtWRI1* with *TCP20* as indicated. (**C**) EMSA demonstrated the TCP20 binding to *proGH3.3* fragments (probe 1 and 2; also see Supplementary Figure S2) in the presence of increasing amount of AtWRI1^1-^^302^ (0.53, 0.71, 1.06, and 2.12 pmol, respectively). The decreasing amount of the protein-DNA complex is indicated by an arrow. (**D**) A proposed model for co-regulation of *GH3.3* expression by TCP20 and WRI1. TCP20 activates *GH3.3* expression by direct binding to *proGH3.3*; however, the activation is attenuated by WRI1 through 1) co-occupation of the promoter and 2) formation of a possible non-DNA binding heterodimer. In any case, the TCP20-AtWRI1 complex exhibits reduced TCP20 target transactivation activity because of the TCP20-AtWRI1 interaction. Hence, in WT, WRI1 fine-tunes TCP20 activation of *GH3.3*, whereas in *wri1-1* that lacks WRI1, TCP20 exhibits stronger activation of *GH3.3* (represented by thicker arrow and increased size of *GH3.3*) compared to WT.

We next examined whether the transactivation activity of TCP20 on *proGH3.3* is attenuated by interaction with AtWRI1. The reporter vector, in which the *LUC* gene expression is driven by *proGH3.3*, was transformed into *N. benthamiana* leaves, alone or in combination with the effectors in which *TCP20* or *AtWRI1* is controlled by the *CaMV* 35S promoter (Figure 3A). While TCP20 significantly activated *proGH3.3*, the activation was greatly reduced when *AtWRI1* was co-expressed (Figure 3B), suggesting that AtWRI1 antagonizes TCP20 upon the interaction. We also conducted a promoter deletion assay to identify the regions in *proGH3.3* critical for TCP20-AtWRI1 mediated expression. We generated three 5′-deletions in *proGH3.3-LUC* (Supplementary Figure S3). The full-length and truncated *proGH3.3-LUC* were transformed into *N. benthamiana* leaves alone, with *AtWRI1*, or with *AtWRI1* and *TCP20*. The 1 kb deletion (from −1940 to −941 bp including one TCP binding motif) caused a significant reduction of LUC activity compared to the full-length promoter (Supplementary Figure S3). The 1.5 kb deletion (from −1940 to −441 bp) did not result in additional loss of activity compared to the 1 kb deletion. When all TCP binding motifs were deleted (−80 bp), less than 10% of the activity remained compared to the full-length promoter. In all cases except the −80 deletion, co-expression of *TCP20* and *AtWRI1* significantly reduced the transactivation of *proGH3.3* by TCP20 (Supplementary Figure S3). These results indicated that the deletion of motif 4 resulted in approximately 70% reduction of TCP20 activation, although the activation of the promoter fragment containing motifs 1–3 was still 15-fold higher than the reporter-alone control. Further removal of motifs 1–3 greatly reduced the activation and abolished the antagonizing effect of AtWRI1. Therefore, while motif 4 contributes most to the TCP20-mediated activation, all four TCP binding motifs contribute to the TCP20 regulation of *proGH3.3*. This conclusion is consistent with the EMSA results showing TCP20 binding to all four TCP binding motifs (Figure 2A).

We demonstrated AtWRI1 attenuating the TCP20 activity on *proGH3.3* possibly by interacting sequestering TCP20. Our previous work shows the binding of AtWRI1 to *proGH3.3*. Additionally, WRI1 did not activate or repress *proGH3.3* in a transactivation assay suggesting that WRI1 likely requires a partner to function [15]. We therefore investigated whether AtWRI1 antagonizes TCP20 from binding to *proGH3.3* in EMSA. The results revealed that AtWRI1 bound to probes 1, 2, and 3 in a TCP binding site-independent manner, as AtWRI1 bound the mutant probes (1M, 2M, and 3M) with equal affinity as the WT probes (Supplementary Figure S4). Since there is no recognizable WRI1 binding motif AW-box found in probes 1–3, AtWRI1 likely recognizes cryptic motifs that are close to the TCP binding motifs. Moreover, upon the addition of an increasing amount of AtWRI1^1-302^, we detected reduced binding of TCP20 to probe 1 and 2 (Figure 3C), but not probe 3 and 4 (Supplementary Figure S5A), in a dose-dependent manner (Supplementary Figure S5B). We therefore speculate that AtWRI1 antagonizes the function of TCP20 through two potential mechanisms: 1) both transcription factors co-occupy *proGH3.3*, resulting in reduced activity of TCP20, and 2) the AtWRI1-TCP20 heterodimer reduces the amount of free TCP20 to activate *proGH3.3* (Figure 3D). In the absence of AtWRI1, e.g., in *wri1-1* mutant, *GH3.3* is significantly upregulated (Figure 3D), leading to the increased production of IAA-Asp conjugates.

TCP20 is known to be involved in cell division, immunity, jasmonic acid biosynthesis, and nitrate foraging [27,28,29]. The regulation of auxin-responsive gene expression in this study defines a new role for TCP20 in Arabidopsis. Prior to this work, it was unclear how AtWRI1 affected auxin homeostasis through regulation of *GH3.3* expression. By demonstration of the direct binding of both AtWRI1 and TCP20 to *proGH3.3* and the protein-protein interaction of the two factors, we elucidate a previously unknown mechanism by which AtWRI1 antagonized the activity of TCP20 on *GH3.3* to modulate auxin homeostasis.

## 3. Materials and Methods

### 3.1. Plant Materials

Arabidopsis and *N. benthamiana* plants were grown in a growth chamber at 23 °C with a photoperiod of 16 h light (100–150 μmol m^−2^ s^−1^ illumination)/8 h dark. Arabidopsis wild-type (Columbia ecotype) was used in this work. Seeds of the *tcp20-2* (SALK_088460) and *tcp20-4* (SALK_041906) mutants, which have been previously described [26], were obtained from the Arabidopsis Biological Resource Center (ABRC). Genotyping assay was conducted to confirm the homozygosity of *tcp20-2* and *tcp20-4* mutants. Reverse transcription-polymerase chain reaction assay was subsequently conducted to verify that the *TCP20* expression was disrupted in *tcp20-2* and *tcp20-4* mutants. Seed sterilization and germination were performed as previously described [30].

### 3.2. Bioinformatic Analysis

In silico analysis of TCP binding sites was performed using AthaMap [31].

### 3.3. Plasmid Construction

Entry constructs subcloning and recombination with destination vectors [Y2H vectors, BiFC vectors (pSITE-nEYFP-C1 and pSITE-cEYFP-C1 [32]), and pEarleyGate binary vectors [33] were via Gateway LR reactions (Life Technologies, Waltham, MA, USA). To generate constructs for epitope-tagged recombinant protein production in *E. coli*, the full-length *AtWRI1* and *TCP20* were sub-cloned into pET41a-GST and pET41a-6×His vectors, respectively, [34]. *AtWRI1^1-302^* was sub-cloned into pNIC28-Bsa4 to produce an N-terminal 6×His-tagged protein (Protein Production Platform, Nanyang Technological University). To generate *proGH3.3:LUC* reporter constructs, various lengths of PCR amplified *proGH3.3* were subcloned into the pGreenII 0800-LUC vector [35]. A list of the primers used for plasmid construction in this study is provided in Table S1.

### 3.4. Yeast Two-Hybrid Assay (Y2H)

For screening Arabidopsis transcription factor library, transcription factors were sub-cloned into the pDEST22 vector (prey) and transformed into yeast strain Y187 (Clontech, San Jose, CA, USA). *AtWRI1* (*AtWRI1^1-306^* and *AtWRI1^58-240^*) variants were sub-cloned into pDEST32 vector (bait) and introduced into yeast strain AH109. The prey and bait were mated and spotted on permissive (-Leu/-Trp) medium. After 3 days, the colonies were streaked onto stringent selective (-Leu/-Trp/-His) medium to screen positive interactions. *TCP20* was subcloned into pDEST22 as the prey and *AtWRI1* variants were subcloned into pDEST32 as the bait. The prey and bait constructs were transformed into Y187 and AH109, respectively. Then the prey and bait were mated and plated on -Leu/-Trp medium. To evaluate the interaction, transformants were streaked onto stringent selective (-Leu/-Trp/-His) medium.

### 3.5. Transient Expression in N. benthamiana, BiFC, and Confocal Microscopy

For BiFC assay, *Agrobacterium tumefaciens* cells carrying the nYFP and cYFP fusion constructs were resuspended in MMA medium (10 mM MgCl_2_, 10 mM MES, 100 µM acetosyringone) to an OD_600_ of 1.2 and adjusted to an OD_600_ of 0.4 before infiltration into *N. benthamiana* leaves. The plasmid pEAQ HT producing the P19 protein was co-infiltrated with other constructs to maintain high expression in *N. benthamiana* leaves [36]. Healthy leaves of *N. benthamiana* plants were infiltrated with *A. tumefaciens* suspensions carrying nYFP and cYFP fusion constructs using a 1 mL blunt-end syringe. After agroinfiltration, plants were placed in a growth chamber. YFP fluorescence signals were detected by a confocal microscope 2–3 days post agroinfiltration. 

### 3.6. Recombinant Protein Production, In Vitro Pull-Down Assays, and EMSA

Recombinant proteins, including the GST-AtWRI1 variants and His-TCP20, were produced in *E coli* strain BL21 (DE3). Protein induction, extraction and purification were conducted as described previously [23,34]. For in vitro pull-down assay, purified His-TCP20 protein was incubated with GST beads coupled with purified GST-AtWRI1 or GST protein in binding buffer [20 mM Tris-HCl (pH 7.4), 150 mM NaCl, 1 mM DTT, 0.1 mM EDTA (pH 8.0), 0.1% IGEPAL CA-630] at 4 °C overnight, followed by washing for 5 times with washing buffer (same as binding buffer). Then the samples were boiled in SDS-PAGE sample buffer at 95 °C for 5 min, followed by SDS-PAGE. The proteins were detected by immunoblot probed with anti-His antibody (Proteintech, Rosemont, IL, USA). EMSA was performed as described previously [34]. In brief, the 5′end biotin-labeled WT probes (1–4) and mutated probes (1M–4M) were used for EMSA. The standard binding reaction (20 µL) contained 0.05 µg/µL poly(dI-dC), 15 mM HEPES-KOH (pH 7.5), 7.5 mM KCl, 0.5 mM EDTA, 5% glycerol, 2 mM dithiothreitol, 1 µg/µL BSA, 2 fmol/µL of the hot DNA probe and ~1 pmol of His-TCP20. The binding competition assays were performed using ~1 pmol of His-TCP20 with addition of His-AtWRI1^1-302^ as described in the figure legend. The reaction mixture was incubated at room temperature for 30 min. The DNA-protein complexes were resolved on 5% (*w*/*v*) non-denaturing polyacrylamide gels and subsequently transferred to nylon membranes. The band shifts were detected by a chemiluminescent nucleic acid detection module (Thermo Fisher Scientific, Waltham, MA, USA).

### 3.7. RNA Extraction, Quantitative Real-Time PCR (qRT-PCR)

Roots from 1-week-old Arabidopsis seedlings grown vertically on the regular growth medium were harvested, immediately frozen in liquid nitrogen, and stored at −80 °C freezer until use for RNA extraction. Total RNA was extracted using the Monarch Total RNA Miniprep Kit (New England Biolabs, Ipswich, MA, USA) following the supplier’s instructions. First-strand cDNA was synthesized using the qScript cDNA Synthesis Kit (Quantabio, Beverly, MA, USA). Quantitative real-time PCR (qRT-PCR) was conducted using Luna Universal qPCR Master Mix (New England Biolabs, Ipswich, MA, USA) according to the supplier’s instructions. *Isopentenyl pyrophosphate:dimethylallyl pyrophosphate isomerase 2 (IPP2)* gene was used as an internal control to normalize the gene expression. The primers used for qRT-PCR is provided in Table S2.

### 3.8. Transient Dual-Luciferase (Dual-LUC) Assays

Transient dual-LUC assays in *N. benthamiana* were conducted as described previously [37,38], with minor modifications. After agroinfiltration, plants were placed in a plant growth chamber, and leaf samples were harvested 3 d after infiltration for the dual-LUC assay using Dual-Luciferase Reporter 1000 Assay System (Promega, Madison, WI, USA). In brief, three leaf discs at agroinfiltration areas (5–6 mm in diameter) were excised and ground in liquid nitrogen to fine powder and homogenized in 100 µL Passive Lysis buffer (Promega, Madison, WI, USA). Subsequently, 5 µL of the extract was mixed with 40 µL Luciferase Assay Buffer, and the firefly LUC activity was measured by a cell imaging multimode plate reader (BioTek Cytation 5, Santa Clara, CA, USA). The reaction was stopped by addition of 40 µL Stop and Glo Buffer (Promega, Madison, WI, USA), and the Renilla (REN) LUC activity was measured. The firefly LUC activity was normalized to the REN LUC activity.

### 3.9. Accession Numbers

Accession numbers are as following: WRI1 (AT3G54320), TCP20 (AT3G27010), GH3.3 (AT2G23170).

## Data Availability

All data supporting the findings of this study are available within the paper and within its Supplementary Materials published online.

## Supplementary Materials

The following supporting information can be downloaded at: https://www.mdpi.com/article/10.3390/plants11030454/s1. Figure S1. Transactivation of the firefly luciferase (LUC) reporter by AtWRI1. Figure S2. In silico analysis of TCP binding sites in *proGH3.3*. Figure S3. The transactivation activity of TCP20 on the *proGH3.3* deletion fragments in *N. benthamiana* leaves. Figure S4. Examination of AtWRI1 binding to *proGH3.3* fragments that are also recognized by TCP20. Figure S5. Effects of AtWRI1 on TCP20 binding to *proGH3.3*. Table S1. Primers used for plasmid construction in this study. Table S2. Primers used for quantitative real-time PCR (qRT-PCR) in this study.
