# Supplementary material for: Functional Antagonism of WRI1 and TCP20 Modulates GH3.3 Expression to Maintain Auxin Homeostasis in Roots
Source: Plants (Basel). 2022 Feb 7;11(3):454. doi: 10.3390/plants11030454 (PMC8840716; doi:10.3390/plants11030454)
Supplement: Supplementary file 1 [file plants-11-00454-s001.zip › plants-1572404-supplementary.pdf]

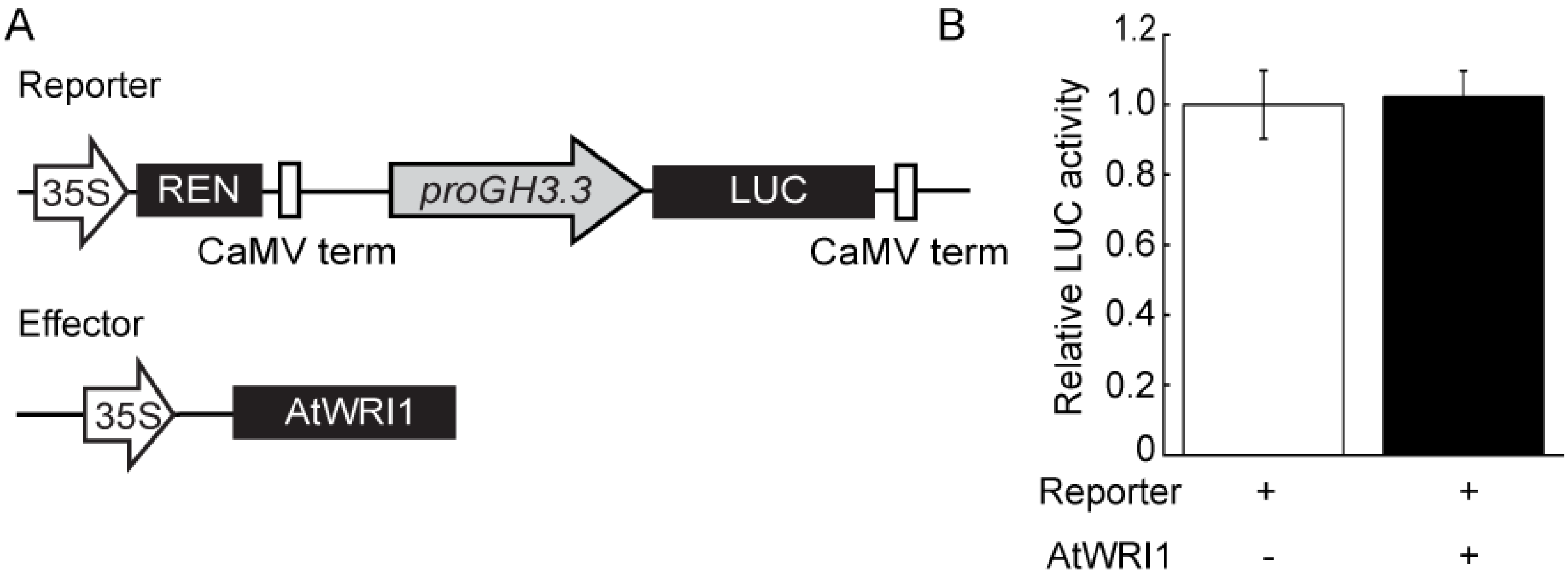

Figure S1. Transactivation of the firefly luciferase (LUC) reporter by AtWRI1. A) Schematic representation of constructs used in a transient expression assay in *N. benthamiana* leaves. The *LUC* reporter gene was driven by a 2kb promoter of *GH3.3* (*proGH3.3*). The *Renilla* luciferase (*REN*) reporter gene was controlled by the *CaMV* 35S promoter. B) Relative reporter activity in *N. benthamiana* leaves transiently expressing the effector and reporter constructs as indicated. The LUC activity was normalized to the REN activity. Results are shown as means ± SE (n=6).

Probe	Relative distance to TSS	Sequence
1	-84	TCTAACGATAACAAACCGAGCCCACTTTTATGTCGACGTGGAATTTGGCT
1M		TCTAACGATAACAAACC*a*AG*aaa*A*a*TTTTATGTCGACGTGGAATTTGGCT
2	-125	ATGTCTGCCCAAAACTAGCCAAAGATTACGTGACCGCGGTCCCTCTTGTCC
2M		ATGTCTGCCCAAAACTAGCCAAAGATTACGTGACCGC*aa*T*aaa*T*a*TTGTCC
3	-170	GACATATCAGTCCCACATGTCTGCCCAAAACTAGCCAAAGATTACGTGACC
3M		GACATATCA*a*T*aaa*A*a*ATGTCTGCCCAAAACTAGCCAAAGATTACGTGACC
4	-944	CTATATATTTTTAATATTTAGGTCCCATTAAATCAGTTTGTGATTTCAGA
4M		CTATATATTTTTAATATTTA*aa*T*aaa*ATTAAATCAGTTTGTGATTTCAGA

Figure S2. *In silico* analysis of TCP binding sites in *proGH3.3*. Putative TCP binding sites were identified by AthaMap (http://www.athamap.de/index.php) in the *GH3.3* promoter region (from 2kb upstream of TSS (transcription start site) to 200bp downstream of TSS). Putative TCP binding sites were highlighted in red. The nucleotide sequences of the wild-type (1-4) and mutated (1M-4M) probes are indicated. The core sequence of TCP binding motif is underlined. The mutations of the TCP binding sequence in the M probes are indicated by italicized small letters.

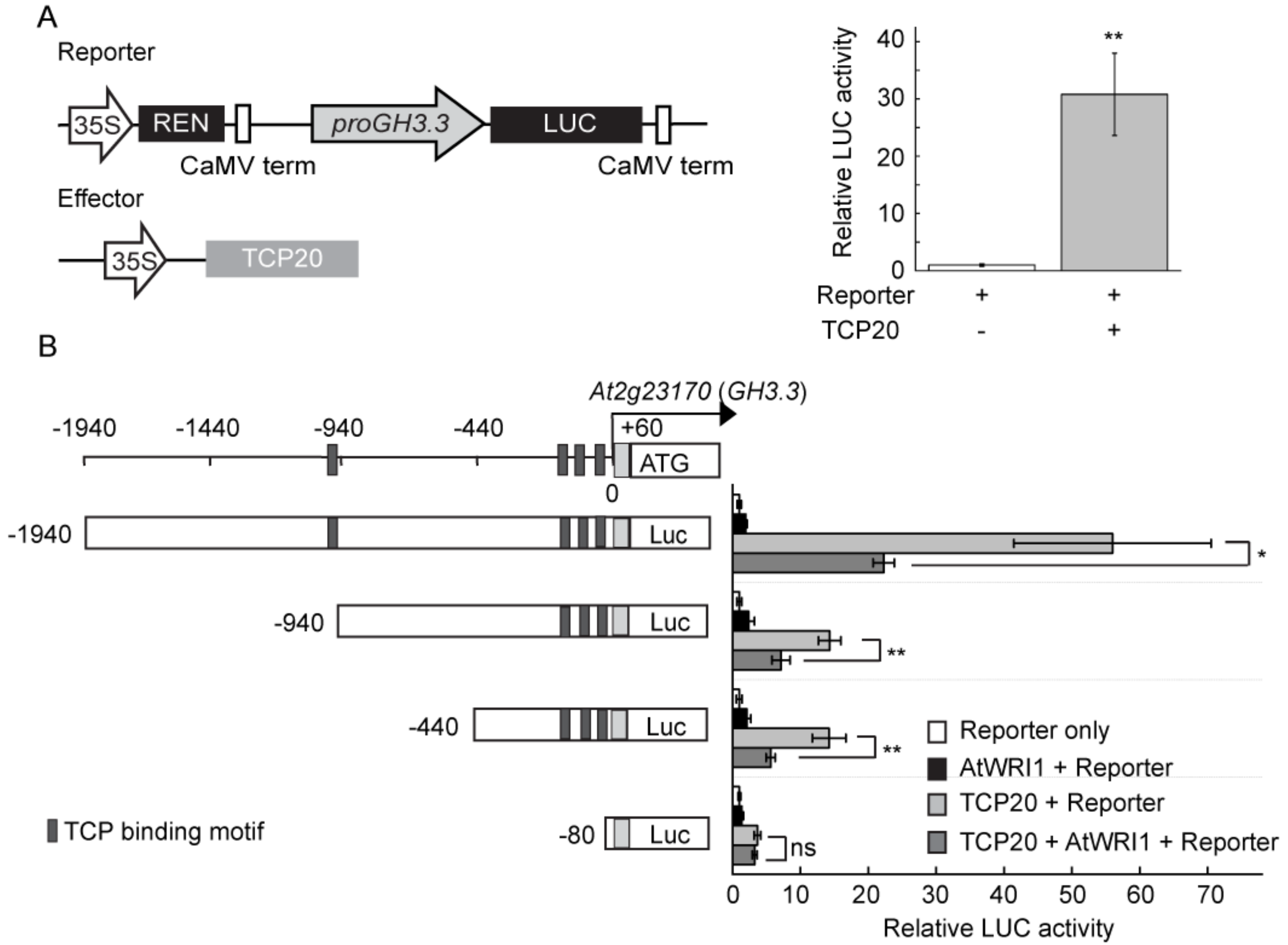

Figure S3. Transactivation of the LUC reporter by TCP20 in *N. benthamiana* leaves. A) Schematic representation of the constructs used in a transient expression assay in *N. benthamiana* leaves. The *LUC* reporter gene was driven by a 2kb *proGH3.3*. The *REN* reporter gene was controlled by the *CaMV* 35S promoter. Relative reporter activity in *N. benthamiana* leaves, infiltrated either using the reporter alone or in combination with the effector, was shown. The LUC activity was normalized to the REN activity. Results are shown as means ± SE (n=5-6). "**" indicates a significant difference (*P*<0.01, one-way ANOVA) between reporter alone and co-transformation of TCP20 and reporter. B) The transactivation activity of TCP20 on the *proGH3.3* deletion fragments in *N. benthamiana* leaves. Co-expression of *AtWRI1* with *TCP20* repressed the transactivation activity of TCP20. Results are shown as means ± SE (n=4-6). '*' and '**' indicate significant differences (P<0.05 and P<0.01, respectively, one-way ANOVA) between sole expression of *TCP20* and co-expression of *AtWRI1* with *TCP20* as indicated. 'ns' represents no statistical significance as determined by one-way ANOVA.

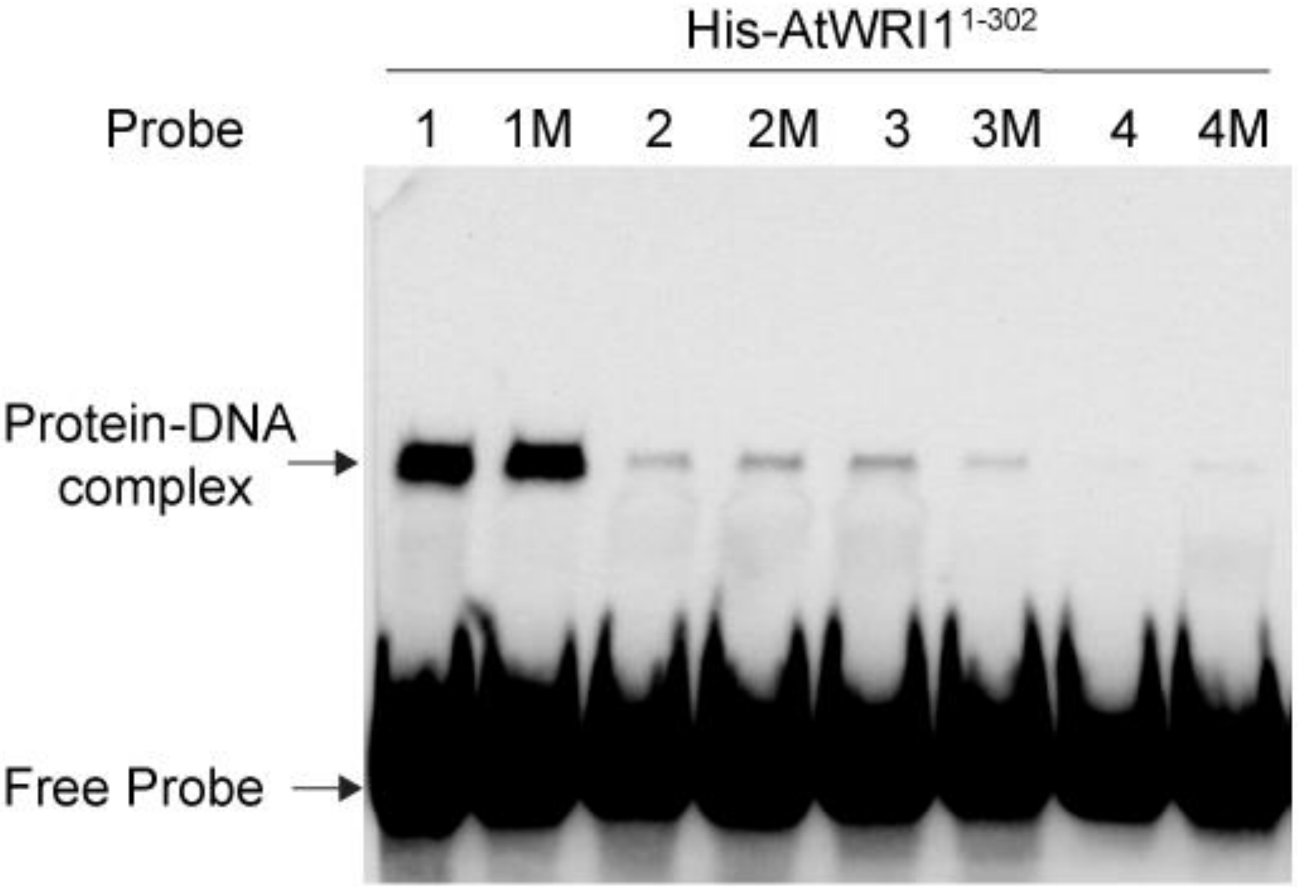

Figure S4. Examination of AtWRI1 binding to *proGH3.3* fragments that are also recognized by TCP20. AtWRI1$^{1\text{-}302}$ binds to probe 1-4 as well as probe 1M-4M (see Figure S2) in EMSA.

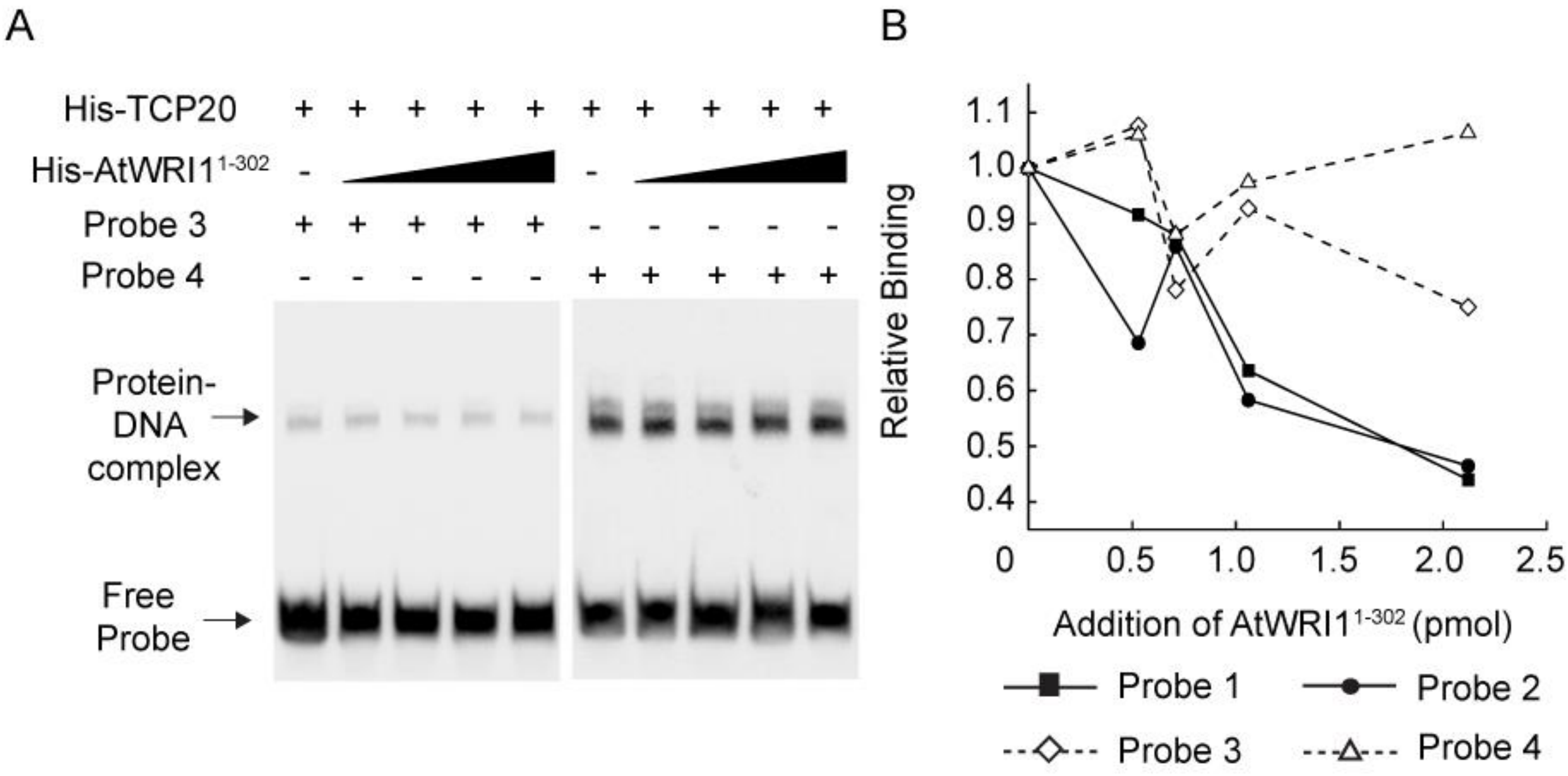

Figure S5. Effects of AtWRI1 on TCP20 binding to *proGH3.3*. A) EMSA showed the binding of TCP20 to *proGH3.3* (probe 3 and 4) in presence of increasing amount of AtWRI1$^{1\text{-}302}$ (0.53, 0.71, 1.06, and 2.12 pmol, respectively). B) Relative binding of TCP20 to probe 1-4 in the presence of increasing amount of AtWRI1$^{1\text{-}302}$ as shown in Figure 3C and Figure S5A.

Table S1. Primers used for plasmid construction in this study.

**Primer Name**	**Sequence 5' to 3'**
AtWRI1-FW	5'-AATGGATCCGGACAATGAAGAAGCGCTTA-3'
AtWRI1-RV	5'-TCCCTCGAGTCAGACCAAATAGTT-3'
$AtWRI1^{58\text{-}240}$-FW	5'-GCAGGATCCATGCTTCTACCCGA-3'
$AtWRI1^{1\text{-}240}$-RV	5'-TAACTCGAGTTACGGGAAAACACC-3'
$AtWRI1^{1\text{-}306}$-RV	5'-TGACTCGAGTCATTCTTCTGAATATCC-3'
TCP20-FW	5'-CGCAGATCTATGGATCCCAAGAACCTA-3'
TCP20-RV	5'-TCCCTCGAGTTAACGACCTGAGCCTTG-3'
$_{pro}$GH3.3 (-1940)-FW	5'-ACTCTCGAGTATTAATTTTTATATCTTATT-3'
$_{pro}$GH3.3 (-940)-FW	5'-ACTCTCGAGATCAGTTTGTGATTTCAGAAT-3'
$_{pro}$GH3.3 (-440)-FW	5'-ACTCTCGAGTCACACACATACTCTAATTCA-3'
$_{pro}$GH3.3 (-80)-FW	5'-ACTCTCGAGTATGTCGACGTGGAATTTGGC-3'
$_{pro}$GH3.3-RV	5'-GCTGGATCCGATTAAAATGGTATTTGTAAG-3'

Table S2. Primers used for quantitative real-time PCR (qRT-PCR) in this study.

**Primer Name**	**Sequence 5' to 3'**
GH3.3-FW	5'-ATCAGTACAAGGTGCCGAGG-3'
GH3.3-RV	5'-AAAGCTGGGCTGAAGTGTGT-3'
IPP2-FW	5'-GAGAAAGGAACTTTGGTTGAAGC -3'
IPP2-RV	5'-GTTTTGTAAGTGTCTCACATATCCC -3'
